# AI-assisted peer review: enhancing evaluation while increasing author workload

**DOI:** 10.1093/haschl/qxag158

**Published:** 2026-07-16

**Authors:** Himel Mondal

**Affiliations:** Departments of Physiology, All India Institute of Medical Sciences, Deoghar, Jharkhand 814117, India

**Keywords:** artificial intelligence, author, peer review

Bauchner and Rivara recently advocated for the integration of artificial intelligence (AI) into editorial and peer-review processes to improve research integrity and manuscript assessment.^1^ While AI-assisted review may enhance the detection of reporting deficiencies and methodological concerns, it may also create an unintended burden for authors.

Traditionally, peer reviewers focused on major methodological, analytical, and reporting issues requiring attention before publication. However, with the increasing availability of large language models (LLMs), reviewers can now generate extensive review reports containing dozens of comments, sub-comments, and stylistic suggestions within minutes.^2^

In a recent submission to a journal related to medical education (with an impact factor of 3.2), we received reports from 4 reviewers. Although we cannot determine whether AI was used, all 4 reports showed characteristics commonly associated with AI-generated text, including extensive bullet-point lists, multiple layers of subheadings, highly structured formatting, and lengthy tables containing numerous comments. One review report contained 2700 words. What might previously have been a concise review identifying key scientific concerns had evolved into a document resembling a detailed audit report.

In an ecosystem where finding peer reviewers is a daunting task,^3^ AI would surely help editors and also reviewers. In addition, detailed peer review undoubtedly improves manuscript quality. However, excessively long reports with very minor issues may create diminishing returns. Authors need to read the full report and discern what are the actionable suggestions. The expectation to provide point-by-point responses to every remark can transform revision into an exhausting administrative exercise rather than a constructive scientific dialog.

Scholarly publishing has long emphasized the importance of sufficiently detailed reviews, but the emergence of AI-generated reports raises a new question: Should there also be an expectation that feedback remains focused, proportionate, and actionable, preferably with a word limit?

## Supplementary Material

qxag158_Supplementary_Data

## Data Availability

No additional data available.
